# Lack of seroprotection against diphtheria in the Austrian population, in light of reported diphtheria cases in Europe, 2022 

**DOI:** 10.2807/1560-7917.ES.2023.28.17.2300206

**Published:** 2023-04-27

**Authors:** Angelika Wagner, Joanna Jasinska, Daniela Schmid, Michael Kundi, Ursula Wiedermann

**Affiliations:** 1 Center of Pathophysiology, Infectiology and Immunology, Institute of Specific Prophylaxis and Tropical Medicine, Medical University Vienna, Vienna, Austria; 2 Center of Pathophysiology, Infectiology and Immunology, Division of Infection Diagnosis and Infectious Disease Epidemiology, Medical University Vienna, Vienna, Austria; 3 Center of Public Health, Institute of Environmental Hygiene, Medical University Vienna, Vienna, Austria

**Keywords:** Diphtheria, vaccine protection, antibody waning, refugees, asylum seekers, booster vaccination

## Abstract

Following an increase in diphtheria cases in Europe since 2022, we retrospectively estimated the prevalence of seroprotection against diphtheria and tetanus in 10,247 Austrian residents (population: 8,978,929) voluntarily tested between 2018 and 2022. Lack of seroprotection against diphtheria was found in 36% compared with 4% against tetanus. The geometric mean antibody concentration against tetanus was 7.9-fold higher compared with that for diphtheria. Raising awareness of regular booster vaccinations against diphtheria in combination with tetanus and pertussis is urgently needed.

Since August 2022, an increase of diphtheria cases among migrants has been observed in Europe [1]. A recent study found that a high proportion of adults in 18 European Union/European Economic Area (EU/EEA) countries did not have protective antibody concentrations against diphtheria [2]. Although no diphtheria cases among the general population in Austria or the EU/EEA have been detected, low seroprotection in the resident community provides an increased risk for disease introduction and outbreaks. Here, we aimed to estimate the prevalence of seroprotection based on the measurement of antibody concentrations as correlate of seroprotection against diphtheria in Austria among 10,247 voluntarily tested individuals between January 2018 and January 2022 to recommend data-based public health measures.

## Seroprotection against diphtheria and tetanus

We included all individuals in Austria who voluntarily provided samples for testing of anti-diphtheria toxoid IgG (DT) and anti-tetanus toxoid IgG (TT) concentrations at the Austrian reference centre for diphtheria, tetanus, and pertussis serology at the Institute of Specific Prophylaxis and Tropical Medicine, Medical University of Vienna between January 2018 and January 2022. In addition, we assessed the extent of DT and TT antibody waning in individuals with antibody results available from a minimum of two time points counted from the latest vaccination. DT and TT antibody concentrations were determined using commercial enzyme-linked immunosorbent assay (ELISA; Binding Site Group Ltd.). Samples with IgG concentrations above the upper limit of quantification (DT: 3 IU/mL, TT: 7 IU/mL) were not further titrated. Antibody concentrations were categorised according to Plotkin et al. [3,4]: non-protective (< 0.01 IU/mL),  inadequately protective (≥ 0.01 to  < 0.1 IU/mL),  adequately protective (≥ 0.1 IU/mL) against diphtheria and tetanus, respectively.

Prevalence of diphtheria and tetanus seroprotection, inadequate seroprotection and non-seroprotection and their multinomial 95% confidence intervals (CI) were estimated by the method of Sison and Glaz [5]. Antibody concentrations were log-transformed and analysed by a generalised linear model, with age categories and sex as independent variables. Geometric mean concentrations (GMC) and 95% CI were calculated based on the least-squares estimates. Waning of antibody titres was analysed by generalised estimating equations model separately for DT and TT antibody concentrations, with age and sex as covariates and time since last vaccination as a within-subject variable.

Between January 2018 and January 2022, 10,247 individuals were included in the analyses of diphtheria seroprotection and 8,034 individuals of tetanus seroprotection (Table 1, see Supplementary Figure S1 for overview of selection of individuals for analyses). For the analysis of antibody waning, 17,468 serum samples from 16,090 individuals, obtained between March 2010 and January 2022 were screened for the availability of vaccination information. Of these, vaccination data and more than one result of anti-DT testing, i.e. from at least two different time points, were available in 89 individuals; in 64 of these individuals, results of anti-TT testing were also available. Overall, test results from 322 samples were analysed for waning of antibody concentrations.

**Table 1:** Individuals with voluntary testing for diphtheria toxoid and tetanus toxoid antibody seropositivity, Austria, January 2018–January 2022 (n = 10,247)

Characteristics	Anti-DT IgG testing n = 10,247		Anti-TT IgG testing n = 8,034	
	n	%	n	%
Age (years)				
≤ 14	722	7.05	615	7.65
15–59	8,443	82.39	6,552	81.55
≥ 60	1,082	10.56	867	10.79
Sex				
Female	6,435	62.80	4,954	61.66
Male	3,785	36.94	3,058	38.06
Unknown	27	0.26	22	0.29

DT: diphtheria toxoid; TT: tetanus toxoid.

**Figure 1:**
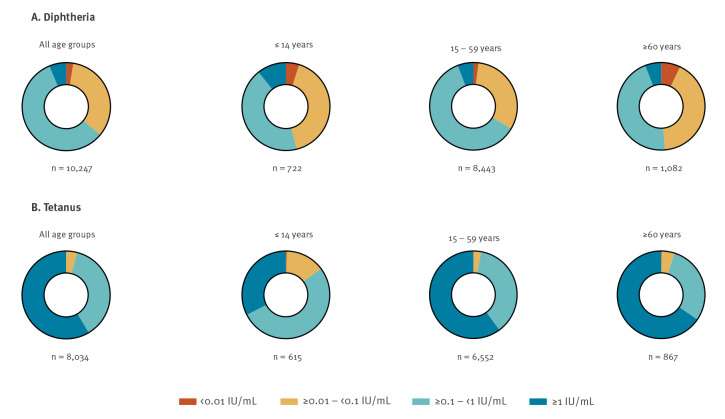
Relative frequency distribution of four categories of diphtheria and tetanus seroprotection in individuals with voluntary testing, Austria, January 2018–January 2022 (n = 10,247)

We found an overall prevalence of seroprotection against diphtheria of 63.96% (95% CI: 62.82–65.09) and against tetanus of 95.99% (95% CI: 95.43–96.48) (Table 2), resulting in a 32.94% (95% CI: 31.78–34.09) prevalence difference (restricted to the 8,034 individuals with both TT and DT antibody results available). In addition, the majority of those tested achieved long-term protection against tetanus but not against diphtheria, as defined by antibody concentrations ≥ 1 IU/mL (Figure 1) [6]. 

**Table 2:** Prevalence of seroprotection and non-seroprotection against diphtheria and tetanus in individuals with voluntary testing, Austria, January 2018–January 2022 (n = 10,247)

Characteristics	Prevalence of seroprotection ≥ 0.1 IU/mL								Prevalence of non-seroprotection < 0.01 IU/mL						Anti-DT GMC n = 10,247		Anti-TT GMC n = 8,034	
	Diphtheria				Tetanus				Diphtheria			Tetanus						
	Total	n	%	95% CI	Total	n	%	95% CI	n	%	95% CI	n	%	95% CI	IU/mL	95% CI	IU/mL	95% CI
All	10,247	6,554	63.96	62.82–65.09	8,034	7,712	95.99	95.43–96.48	264	2.58	2.23–2.98	4	0.05	0.02–0.15	0.14	0.13–0.14	1.10	1.07–1.13
Age (years)																		
≤ 14	722	389	53.88	49.42–58.27	615	524	85.20	81.45–88.31	35	4.85	3.26–7.14	2	0.33	0.07–1.50	0.11	0.10–0.12	0.49	0.45–0.54
15–59	8,443	5,609	66.43	65.19–67.65	6,552	6,364	97.13	96.59–97.58	152	1.80	1.49–2.18	0	0.00	0.00–0.09	0.15	0.14–0.15	1.17	1.13–1.20
≥ 60	1,082	556	51.39	47.75–55.01	867	824	95.04	92.96–96.53	77	7.12	5.46–9.22	2	0.23	0.05–1.07	0.09	0.08–0.10	1.27	1.17–1.37
Sex																		
Female	6,435	3,930	61.07	59.61–62.52	4,954	4,739	95.66	94.91–96.30	191	2.97	2.50–3.52	2	0.04	0.01–0.19	0.12	0.12–0.13	1.01	0.98–1.05
Male	3,785	2,604	68.80	66.97–70.57	3,058	2,952	96.53	95.65–97.24	71	1.88	1.42–2.48	2	0.07	0.01–0.30	0.16	0.16–0.17	1.27	1.22–1.33

CI: confidence interval; DT: diphtheria toxoid; GMC: geometric mean concentration; TT: tetanus toxoid.

Antibody titres were interpreted as non-protective (antibody concentration of < 0.01 IU/mL) and protective (antibody concentration of ≥ 0.1IU/mL).

Antibody titres against (A) diphtheria and (B) tetanus were interpreted as non-protective (antibody concentration of < 0.01 IU/mL (red)) inadequately protective (antibody concentration of ≥ 0.01 −  < 0.1 IU/mL (yellow)), adequately protective (antibody concentration of ≥ 0.1 – < 1 IU/mL (light blue)) and long-term protective (antibody concentration ≥ 1 IU/mL (dark blue)).

**Figure 2:**
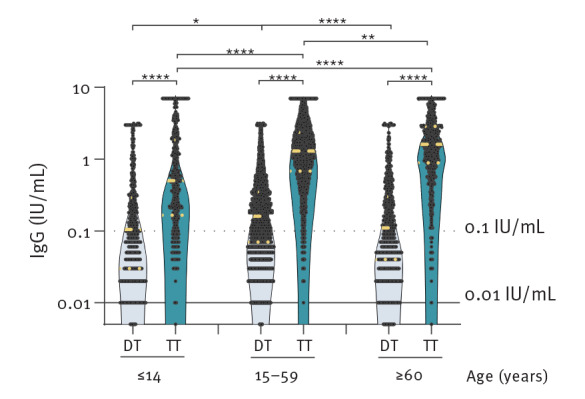
Diphtheria toxoid (n = 10,247) and tetanus toxoid (n = 8,034) geometric mean antibody concentrations by age groups in individuals with voluntary testing, Austria, January 2018–January 2022

DT: diphtheria toxoid; TT: tetanus toxoid.

The prevalence of diphtheria seroprotection in male participants was found to be 1.13 times (95% CI: 1.09–1.17) higher than in female participants (Table 2). The diphtheria seroprotection differed significantly (p < 0.0001) between the three age groups, with the lowest seroprotection prevalence in those aged  60 years and older (51.39%; 95% CI: 47.75–55.01), followed by those aged 14 years and under (53.88%; 95% CI: 49.42–58.27); those between 15 and 59 years of age had the highest prevalence of seroprotection (66.43%; 95% CI: 65.19–67.65) (Table 2, Figure 1). The anti-TT GMC was 7.9-fold higher compared with the anti-DT GMC (anti-DT GMC: 0.14, 95% CI: 0.13–0.14; anti-TT GMC: 1.10, 95% CI: 1.07–1.13); p < 0.0001). Those 60 years and older showed the lowest GMCs of anti-DT, while those aged between 15–59 years had the highest (Table 2, Figure 2).

Median values (yellow line) and interquartile range (yellow dotted line) are represented. Levels of significance in a Kruskal–Wallis test are indicated by asterisks: **** p < 0.0001, ** for p < 0.01 and * for p < 0.05.

## Diphtheria and tetanus toxoid antibody waning

The DT antibody concentrations declined with an average percentage change of 2.9% (95% CI: 0.6–5.0; p < 0.001) in the 89 individuals. The DT antibody concentrations decreased with age, with a percentage change of 16.5% (95% CI: 8.0–24.1; p < 0.001) for each 10-year increase in age (data not shown; of note: individuals were not split up into age groups but entered with their exact age).

The estimated initial GMC of TT antibodies following the latest vaccination was 20.6-fold higher than that of the DT antibodies (GMCs TT: 3.92 IU/mL vs GMCs DT: 0.19 IU/mL). The TT antibody concentrations decreased with an average annual percentage change of 6.9% (95% CI: 4.6–9.1; p = 0.012, data not shown). However, because of the high initial TT antibody concentrations, the average decline of antibodies was estimated not to reach the threshold of inadequate seroprotection of 0.1 IU/mL earlier than 50 years after the last vaccination in the subgroup of the 64 individuals.

## Discussion

After the introduction of general childhood vaccination against diphtheria in Austria in 1946, the occurrence of the disease has remarkably decreased by the end of the 1960s. Only sporadic cases of infection with toxigenic *Corynebacterium diphtheriae* were reported until recently [7], despite an insufficient coverage of primary childhood vaccination (85%, rank 141/192 countries) [8-10]. Up to 24 January 2023, 242 imported cases of diphtheria have been reported by eight EU/EEA countries, including 72 in Austria, all among recently arrived migrants [11]. In Austria, one fatal case of respiratory diphtheria was reported in a migrant from outside of the Europe. Thus far, the European Centre for Disease Prevention and Control (ECDC) has no indication of a spread from the currently affected groups to the general population. However, an increased risk of exposure to *Corynebacterium species* in individuals in contact with the reception centres or their residents lacking sufficient protection against diphtheria needs consideration. Therefore, knowledge on the seroprotection prevalence in the population is crucial for potential implementation of public health measures, such as vaccination campaigns for disease control. 

We observed a low prevalence of seroprotection against diphtheria in the tested population, which raises the question whether this is due to missed booster vaccinations, early antibody waning or low vaccine-induced antibody levels. The lower DT vs TT antibody levels might be due to a reduced DT content in the adult booster vaccine formulation compared with the infant formulation [12,13]. In addition, the considerably higher tetanus antibody titres could be a result of a preferred use of monovalent tetanus vaccines (no longer available) in emergency care settings in the past. The DT antibody levels decreased more slowly than the TT antibody levels, but because the levels started from a lower average concentration, participants’ diphtheria antibody levels were more likely to fall below the protective level. These findings, albeit based on a small sample, may imply that the national recommendation for regular boosters with combined vaccines, i.e. diphtheria/tetanus/pertussis ± polio vaccines every 10 years until the age of 60 years and thereafter every 5 years, is not followed [14]. The fact that 36% of individuals actively requesting testing of their DT antibody levels lack adequate seroprotection against diphtheria requires immediate attention, especially given the assumption that seroprotection level in the general population may be even lower.

Currently, the most important interventions are (i) to provide information and easy access to primary and booster vaccination to populations at risk, such as migrants from regions with low vaccination coverage due to disruption of medical service in their home countries, (ii) to improve the awareness of high coverage for primary and regular booster vaccinations so herd immunity is achieved in the local population and (iii) to use booster vaccinations with monovalent diphtheria vaccines (as was previously available) to avoid side effects because of unnecessarily increased TT antibodies, or to consider a higher diphtheria toxoid dose for the adult booster vaccine formulation; the latter may need further evidence from larger studies 

Vaccination and medical history were incomplete for the whole study population because of lack of information on the referral sheets. Although we do not know the intention of the individuals for whom we analysed the anti-TT and anti-DT antibody concentrations, experience indicates that many wish to have their level of seroprotection tested before they decide whether to receive a booster vaccination. For some individuals, the sera were sent in by their treating physicians because of an underlying disease. Thus, the study population is not necessarily representative of the general population and might have a higher health awareness. Thus, we suggest that the observed prevalence of seroprotection is probably higher than in the general population, which may be unaware of the need for regular booster vaccinations.

## Conclusions

Our findings, combined with a low coverage of 85% for primary diphtheria vaccination in children in Austria, signal that the detection of diphtheria cases among migrants coincides with an inadequately immunised resident population. In order to protect both migrants and the resident population from diphtheria infections, there is a need to improve seroprotection by vaccination in both groups.

## Supplementary Data

93000 Supplement
