# Supplementary material for: Lack of seroprotection against diphtheria in the Austrian population, in light of reported diphtheria cases in Europe, 2022
Source: Euro Surveill. 2023 Apr 27;28(17):2300206. doi: 10.2807/1560-7917.ES.2023.28.17.2300206 (PMC10283473; doi:10.2807/1560-7917.ES.2023.28.17.2300206)
Supplement: Supplement [file 23-00206_WIEDERMANN_SUPPLEMENT.pdf]

“This supplementary material is hosted by Eurosurveillance as supporting information alongside the article “**Diphtheria cases among asylum seekers/migrants in Austria collide with lack of seroprotection against diphtheria in the resident population”** on behalf of the authors who remain responsible for the accuracy and appropriateness of the content. The same standards for ethics, copyright, attributions and permissions as for the article apply. Eurosurveillance is not responsible for the maintenance of any links or email addresses provided therein.

Supplementary Figure S1. Study inclusion of individuals requesting voluntary testing for diphtheria toxoid and tetanus toxoid antibody concentrations, Austria, March 2010-January 2022 (n=16,090)

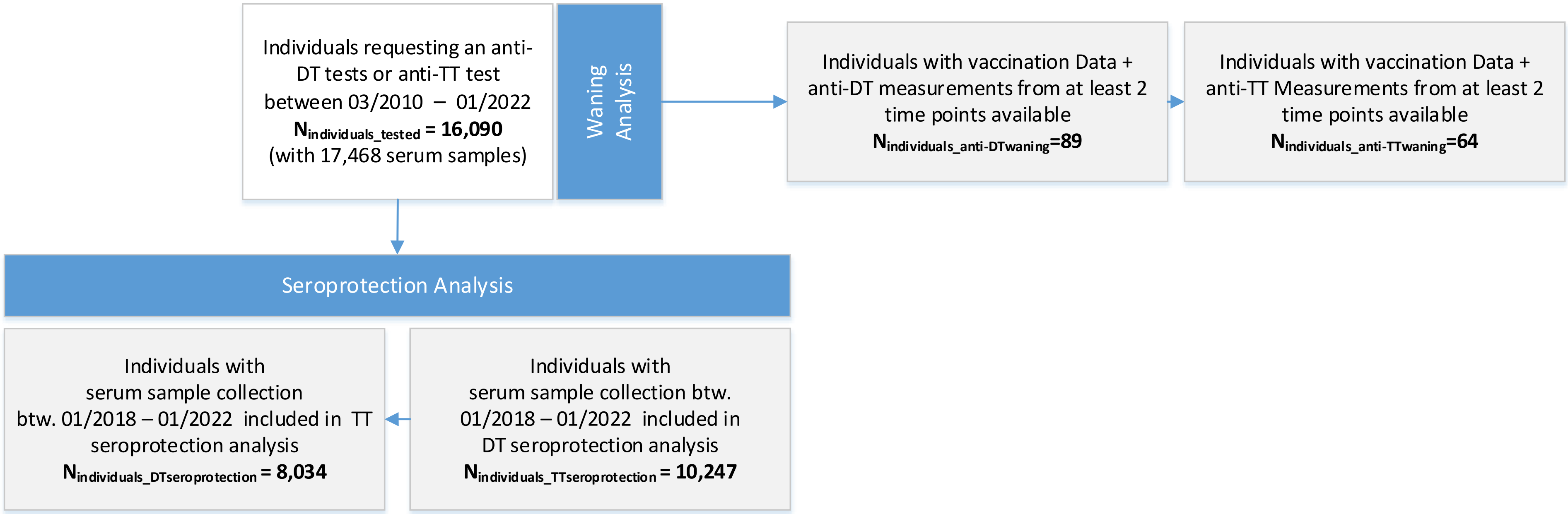

DT: diphtheria toxoid; TT tetanus toxoid
